# Supplementary material for: The interactome of intact mitochondria by cross-linking mass spectrometry provides evidence for coexisting respiratory supercomplexes
Source: Mol Cell Proteomics. 2017 Dec 8;17(2):216–32. doi: 10.1074/mcp.RA117.000470 (PMC5795388; doi:10.1074/mcp.RA117.000470)
Supplement: Supplemental Data [file supp_RA117.000470_133922_0_supp_23978_fzffwf.zip › spectra_annotation/mito_spectra_annotation/483-1-5-1-1-1.pdf]

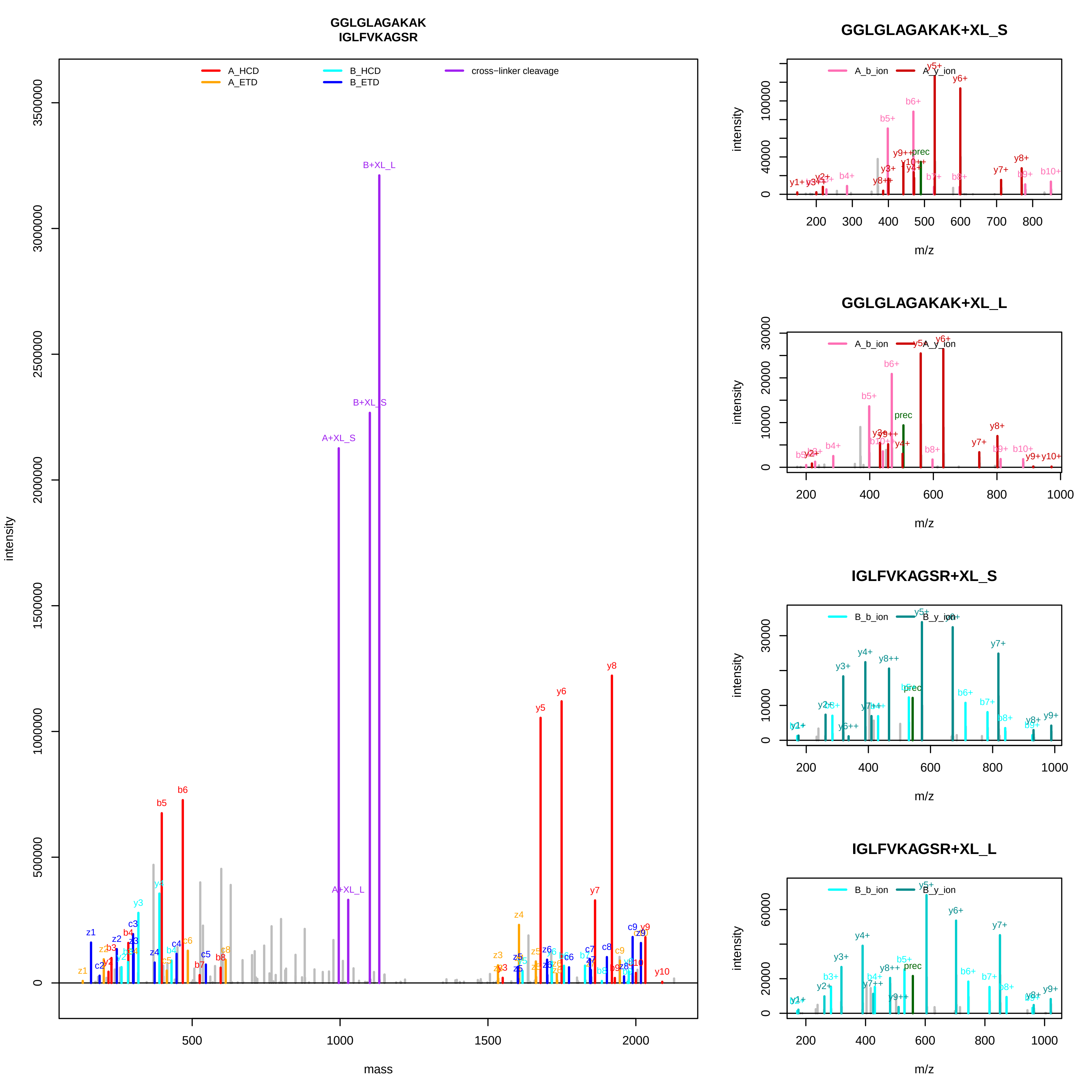

GGLGLAGAKAK
IGLFVKAGSR
A_HCD
A_ETD
B_HCD
B_ETD
cross−linker cleavage
intensity
mass
0
500000
1000000
1500000
2000000
2500000
3000000
3500000
500
1000
1500
2000
B+XL_L
B+XL_S
A+XL_S
A+XL_L
b5
b6
y5
y6
y7
y8
y9
y10
z4
y3
y4
GGLGLAGAKAK+XL_S
A_b_ion
A_y_ion
intensity
m/z
0
40000
100000
200
300
400
500
600
700
800
y5+
y6+
b6+
b5+
prec
y8+
y7+
b10+
b9+
b4+
y3+
GGLGLAGAKAK+XL_L
A_b_ion
A_y_ion
intensity
m/z
0
10000
20000
30000
200
400
600
800
1000
y6+
b6+
b5+
prec
y8+
y7+
b8+
b4+
b10+
IGLFVKAGSR+XL_S
B_b_ion
B_y_ion
intensity
m/z
0
10000
30000
200
400
600
800
1000
y5+
y6+
y7+
y4+
y8++
y3+
b6+
b7+
prec
y9+
IGLFVKAGSR+XL_L
B_b_ion
B_y_ion
intensity
m/z
0
20000
60000
200
400
600
800
1000
y5+
y6+
y7+
y4+
y3+
b5+
b6+
b7+
prec
y9+
